# Alzheimer’s Disease and Healthy Aging Indicators Cognitive Decline

**DOI:** 10.1002/alz.084491

**Published:** 2025-01-09

**Authors:** Hamna Khuld

**Affiliations:** ^1^ Columbia University, New York, NY USA

## Abstract

**Background:**

In the United States and Puerto Rico, cognitive decline and resulting Alzheimer’s disease are major public health concerns for older adults. According to the Alzheimer’s Association (2021), by 2050, people ages 65 and above with Alzheimer’s are estimated to grow to 12.7 million—rising in prevalence and severity. The rise in Alzheimer’s prevalence is attributed to genetic, environmental, and lifestyle factors—leading to memory, judgment, language, and behavior problems. This research examines whether there is an association between race and cognitive decline as indicated by adults ages 50 and above in the United States and Puerto Rico.

**Method:**

The Alzheimer’s Disease and Healthy Aging Indicators Cognitive Decline study is an ecological study that documents cognitive decline or memory loss among adults ages 50 and above. The data was collected from 2015 to 2019 by the Center for Disease Control, under the Behavior Risk Factor Surveillance System (BRFSS). The surveys documented cognitive decline or memory loss experienced by 16,799 older adults by age, race, sex, and state of residency.

**Result:**

The main findings examine the association of cognitive decline by comparing races, using White, non‐Hispanic as the reference group. This analysis shows that Hispanic vs White has the highest OR of 10.35 and 95% CI of 3.128 to 34.256. Black, non‐Hispanic vs White, non‐Hispanic have the second‐highest OR at 3.130 with 95% CI at 1.277 to 7.674.

Association between cognitive decline and race showed a negative inverse relationship–cognitive decline (BRFSSPRCTG) decreases depending on the race. Races such as Native Am/Alaskan Native, Hispanic, and Black, White are statistically significant. However, Asian/Pacific Islanders had on average 0.67 standard error, which is not statistically significant (alpha = 0.05).

Age and its association with participants' state of residency shows that Maryland had the highest percentage of cognitive decline by either age group–ages 50‐64 years are 4.76%, and ages 65 and older are 4.50% likely to experience cognitive decline.

**Conclusions:**

In conclusion, race is associated with cognitive decline depending on age and state of residency. Further research is needed because there was missing information on participants' education, income, zip code, socioeconomic status, etc., which may contribute to cognitive decline.

